# Major Depression Is Not Associated with Blunting of Aversive Responses; Evidence for Enhanced Anxious Anticipation

**DOI:** 10.1371/journal.pone.0070969

**Published:** 2013-08-08

**Authors:** Christian Grillon, Jose A. Franco-Chaves, Camilo F. Mateus, Dawn F. Ionescu, Carlos A. Zarate

**Affiliations:** 1 Section on Neurobiology of Fear and Anxiety, National Institute of Mental Health, National Institutes of Health, Bethesda, Maryland, United States of America; 2 Experimental Therapeutics and Pathophysiology Branch, National Institute of Mental Health, National Institutes of Health, Bethesda, Maryland, United States of America; University of Gent, Belgium

## Abstract

According to the emotion-context insensitivity (ECI) hypothesis, major depressive disorder (MDD) is associated with a diminished ability to react emotionally to positive stimuli and with blunting of defensive responses to threat. That defensive responses are blunted in MDD seems inconsistent with the conceptualization and diagnostic nosology of MDD. The present study tested the ECI hypothesis in MDD using a threat of shock paradigm. Twenty-eight patients with MDD (35.5±10.4 years) were compared with 28 controls (35.1±7.4 years). Participants were exposed to three conditions: no shock, predictable shock, and unpredictable shock. Startle magnitude was used to assess defensive responses. Inconsistent with the ECI hypothesis, startle potentiation to predictable and unpredictable shock was not reduced in the MDD group. Rather, MDD patients showed elevated startle throughout testing as well as increased contextual anxiety during the placement of the shock electrodes and in the predictable condition. A regression analysis indicated that illness duration and Beck depression inventory scores explained 37% (p<.005) of the variance in patients’ startle reactivity. MDD is not associated with emotional blunting but rather enhanced defensive reactivity during anticipation of harm. These results do not support a strong version of the ECI hypothesis. Understanding the nature of stimuli or situations that lead to blunted or enhanced defensive reactivity will provide better insight into dysfunctional emotional experience in MDD**.**

## Introduction

Major depressive disorder (MDD) is a devastating psychiatric condition of mood dysregulation characterized by disturbance in positive and negative emotional experiences. Elucidating the nature of such disturbance is a necessary step toward clarifying pathophysiology and improving diagnostic, treatment, and prevention efforts. While dysregulated affect is a hallmark of MDD, the nature of such dysregulation remains to be characterized. It has been suggested that MDD is characterized by blunted emotional response not only to positive stimuli [1], [2], [3] but, perhaps more surprisingly, to negative stimuli [4]. Indeed, Rottenberg et al [5] proposed the emotion-context insensitivity (ECI) hypothesis, according to which MDD is associated with diminished reactivity to both rewarding and threatening stimuli. Although the ECI hypothesis has recently been supported in a meta-analysis [6], the hypothesis that MDD shows reduced defensive response is inconsistent with substantial neurocognitive evidence of hyperactive aversive emotional responding [7], [8] and with the theoretical conceptualization that depression and anxiety disorders share a common distress factor of heightened affective negativity [9]. Such a distress factor leads to an alternative to the ECI hypothesis, the negative potentiation hypothesis [6], which predicts exaggerated not blunted response to aversive stimuli. The negative potentiation hypothesis is supported by evidence that the amygdala can be hyper-reactive in MDD [10], [11] together with findings of enhanced fear conditioning in this condition [12]. In addition, high trait anxiety/neuroticism is a vulnerability marker for depression [13] and depressed individuals exhibit substantially high levels of anxiety/neuroticism, which should also lead to exaggerated aversive responding.

The hypothesis that depression is a state of reduced defensive reactivity is increasingly becoming influential and has important implications. Conceptually, it raises issue regarding the nature of negative affectivity and anxiety in depression. Specifically, reduced defensive engagement in MDD suggests that anxiety in depression is different from anxiety in anxiety disorders. From a therapeutic viewpoint, it has been argued that treatment that increases emotional response to positive and negative stimuli would be beneficial [6] and that treatment aimed at reducing defensive reactivity may not be optimal [14]. Clearly, the nature of defensive reactivity in MDD needs clarification.

Support for the ECI hypothesis has been provided by startle studies. The startle reflex is a cross-species reflex that is potentiated by aversive states [15]. It is a useful tool to investigate defensive reactivity; potentiation of the startle reflex reflects the priming of limbic system-mediated activation of defensive mechanisms. Consistent with the ECI hypothesis, startle studies in MDD have found reduced or lack of startle potentiation during the processing of negative pictures or videos. This blunting of defensive reflexes has been reported in severely depressed individual as well as in subclinical depression and in patients on and off medications [16], [17], [18], [19], [20], [21]. Additionally, emotional blunting may be more pronounced when depression is comorbid with anxiety [14], [22], [23].

While there is clear evidence that MDD can be associated with emotional bluntness, the ECI hypothesis may be an over-generalization of the findings. One possible explanation for the finding of emotional bluntness in MDD, at least in startle studies, is that it reflects withdrawal from the environment rather than a general blunting of defensive mechanisms. Indeed, withdrawal from the environment may be an important adaptive mechanism for MDD [24]. But it may not be possible to disengage from all aversive stimuli or situations. For example, it may be easier to disengage from stimuli that do not pose a direct threat compared to stimuli that cause physical harm [25]. Blunting of startle potentiation in depression has been shown using procedures, such as watching emotional pictures (e.g., International Affective Picture System [26]) or imagery [23], that use hypothetical situations without physical threat. Further, these procedures do not evoke strong defensive responses and require subjects’ attentional involvement, which may be diminished compared to healthy controls. This raises the question as to whether the emotional numbness of MDD patients generalizes to conditions where strong defensive responses are evoked by *actual* physical threat such as shocks. In fact, a recent study found no reduced startle potentiation in MDD during anticipation of shock [27]. However, the MDD participants were on active psychiatric medications, preventing firm interpretation of the findings.

In both humans and animals, distinct types of defensive responses can be evoked in anticipation of noxious stimuli; a phasic fear response to proximal threat and a more sustained anxiety state induced by contextual, distal, or unpredictable stressors, the former being mediated primarily by the amygdala and the later by the bed nucleus of the stria terminalis (BNST) [15]. We have reported that individuals with posttraumatic stress disorder (PTSD) or panic disorder show a sustained increase in ‘baseline’ startle by contextual threatening cues (e.g., experimental room, shock electrodes) in experiments in which shocks are administered, a response that we have attributed to the distal shock threat generalizing to the environment (context-potentiated startle) [28]. We have developed a paradigm to model phasic and sustained defensive responses in a more controlled manner. It consists in examining startle potentiation during anticipation of predictable and unpredictable aversive events [29]. In the predictable condition, the aversive stimulus is signaled by a threat cue, evoking a phasic fear-potentiated startle response. In the unpredictable condition, the aversive stimulus is not signaled, resulting in a more sustained anxiety-potentiated startle response (note that, consistent with the animal literature, the no cue period of the predictable condition also induce a sustained anxiety-potentiated startle response, but of smaller magnitude). We recently showed that individuals with PTSD or panic disorder exhibit normal fear-potentiated startle during predictable aversive anticipation, but show increased anxiety-potentiated startle during unpredictable aversive anticipation [30], [31]. However, in contrast to our earlier studies, we did not find increased ‘baseline’ startle (i.e., contextual anxiety), probably because we used stimuli less aversive than shocks (a combination of loud noises, scream, and strong puff of air to the neck, which do not evoke strong contextual anxiety).

The present study examined defensive responses in non-medicated individuals with MDD during threat of predictable and unpredictable shocks. The ECI model predicts lack of startle potentiation in MDD. Finding robust startle potentiation during shock anticipation in MDD would contradict the model. We expected not only that the ECI hypothesis would not be supported, but also that defense reactivity in MDD would be more consistent with the negative potentiation hypothesis (i.e., MDD associated with increased defensive reactivity). This latter hypothesis would be supported by findings that MDD individuals show enhanced sensitivity to the threatening experimental context, resulting in increased in baseline startle and/or enhanced startle responses to contextual cues (i.e., shock electrodes) as found in individual at-risk for MDD [32] and in PTSD [28], or in response to predictable or unpredictable shocks.

Blunting of the affective modulation of startle has been associated more frequently with increased negative affectivity as assessed with the Beck Depression Inventory [33], illness chronicity [19], [23], and comorbid anxiety disorders [14], [22], [23]. A secondary aim was to examine whether any of these variables were associated with a potential blunting of defensive responses in MDD.

## Methods

### Participants

Twenty-eight seeking-treatment but medication-free inpatients (17 women; mean ± SD age, 35.5±10.4 years; range 19–55 years) with MDD and 28 age- and sex-matched healthy controls (17 women; 35.7±10.4 years; range 23–53 years) participated in the study. Before inclusion, all MDD patients were clinically assessed by trained psychiatrists at the Experimental Therapeutics and Pathophysiology Branch of the NIMH. This examination included the Structural Clinical Interview for DSM-IV diagnosis (SCID) [34], the self-rated Spielberger State-Trait Anxiety Inventory (STAI) [35], and the self-rated Beck Depression Inventory (BDI) [33]. All patients met DSM-IV criteria for MDD and 17 patients also had a comorbid diagnosis of lifetime anxiety disorder. Healthy controls were screened using SCID by trained psychologists at the NIMH. They had no current or past psychiatric diagnosis and did not have first-degree relative with mood or anxiety disorders. All subjects had a negative urine screen. Patients had significantly higher STAI [35] scores, compared to controls (State anxiety: 41.8±8.7 versus 26.7±7.4; t(54) = 10.7, p<.0009; trait anxiety: 51.7±8.7 versus 28.3±5.2; t(54) = 9.0, p<.0009). Patients’ mean BDI score was 29.2 (±10.1). After complete description of the study to the subjects, written informed consent was obtained. The study was conducted in accordance with the Declaration of Helsinki and was approved by the NIH Institutional Review Board.

### Procedure

**Figure 1:**
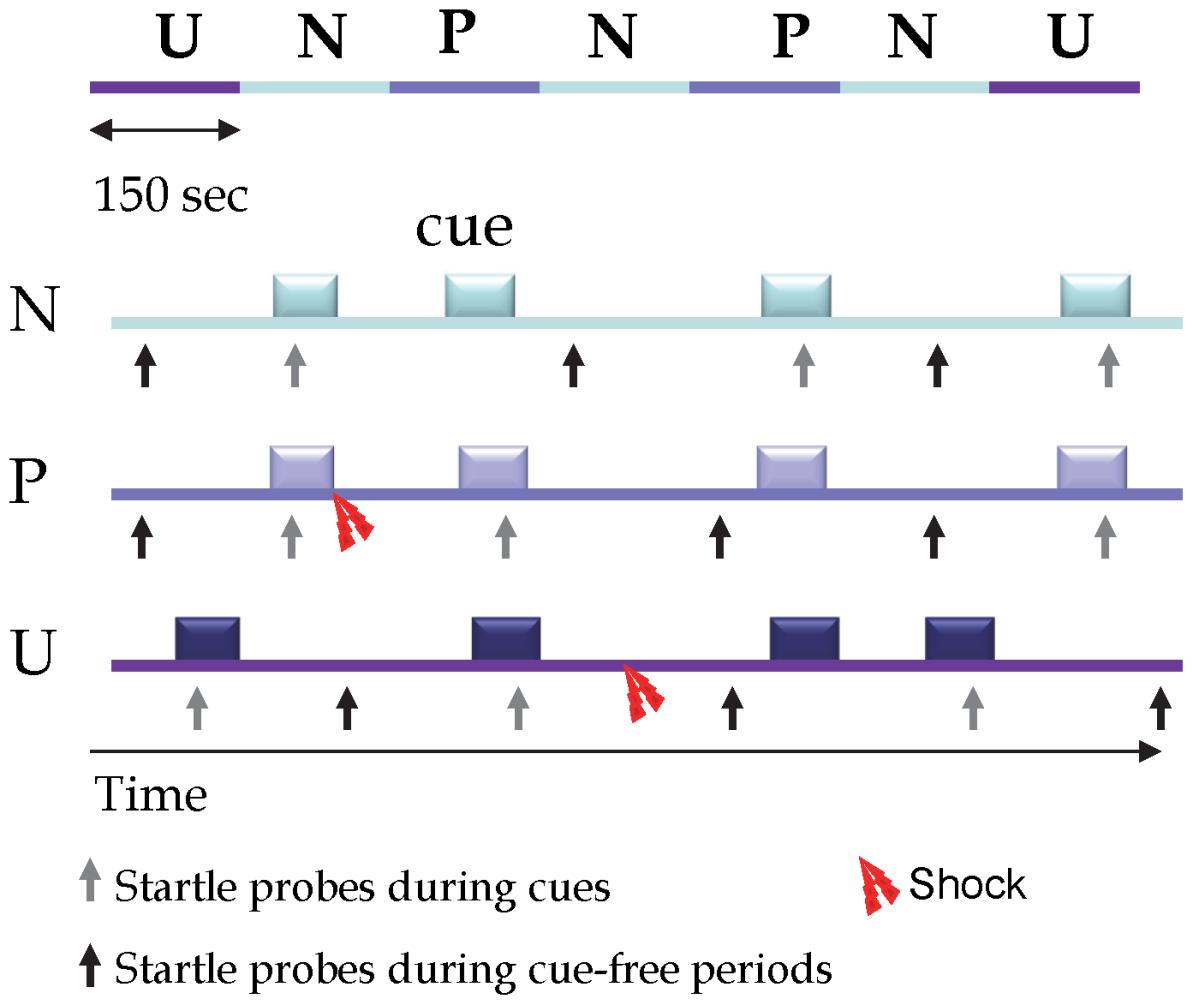
Schematic representation of sequences of stimulus presentation during each condition in one block of the NPU-threat test. The upper part of the figure represents a complete block, including two P (predictable), two U (unpredictable) and three N (no shock) conditions (order UNPNPNU as shown or PNUNUNP). The lower part shows each condition, including cues (8-s duration), startle probes presented during cues (grey arrow pointing up) or during cue-free periods (dark arrow pointing up), and shocks. Adapted from reference 31.

The procedure is described in details in a methodology article [29] and was similar to that of recent investigations testing the effect of anxiolytics on startle potentiation during shocks anticipation [36], [37]. It consists in examining startle reactivity during three conditions, no shock, predictable shock, and unpredictable shock (NPU-threat test). Briefly, after attachment of the eyeblink electrodes, nine startle stimuli were delivered every 18–23 s to habituate the startle response (startle habituation 1) and to examine potential group difference in startle reactivity. The shock electrodes were then attached to the wrist and a shock work up procedure was started to set the shock intensity at a mildly painful level. Next, participants were given explicit instructions regarding the conditions under which the shocks were administered. There were three 150-sec conditions (Fig. 1): no-shock (N); predictable shock (P); and unpredictable shock (U) (see [29] for schematic description). An 8-sec duration cue was presented four times in each condition. The cues were different colored geometric shapes for each condition (e.g., green circle for N, red square for P). The cues signaled the possibility of receiving an aversive stimulus in the P condition. They had no signal value in the N and U conditions. During the experiment, the following written instructions were continuously displayed on a monitor facing the participants: “no shock” (N), “shock only during shape” (P), or “shock at any time” (U). Each participant was presented with two blocks of three N, two P, and two U with the following orders P N U N U N P or U N P N P N U, with the two orders being counterbalanced within each group. Two shocks were administered in each individual P and U condition for a total of 8 shocks during the session. The shocks were delivered at the end of the cue in the P condition and in the absence of a cue in the U condition.

Four habituation startle stimuli were delivered at the beginning of each block (startle habituation 2 and habituation 3, for block 1 and 2, respectively). This was followed by six startle stimuli in each individual condition (N, P, and U), three during cue-free periods (i.e., inter-trial intervals or ITI) and one during three of the four cues, 5–7 sec following cue onset. The mean inter-startle interval was 21 s (range 18–25 s) and no startle stimulus was delivered less than 8 sec after an aversive stimulus in order to avoid potential short-term sensitization of startle.

After each block, subjects were asked to rate retrospectively their anxiety level in the presence and absence of the cue in each condition (N, P, U) on an analog scale ranging from 0 (not at all anxious) to 10 (extremely anxious).

### Stimuli and Physiological Responses

Stimulation and recording were controlled by a commercial system (Contact Precision Instruments, London, England). The acoustic startle stimulus was a white noise (40-ms duration, 103-dB (A)) presented through headphones. The startle/eyeblink electromyographic (EMG) signal was recorded with electrodes under the left eye, and was then digitized (1000 Hz) and amplified (bandwidth 30–500 Hz).

### Data Reduction and Analysis

Peak blink amplitude was determined in the 20–100-ms time frame following stimulus onset relative to baseline (average baseline EMG level for the 50 ms immediately preceding stimulus onset). Startle magnitude was analyzed using raw scores and standardized scores using within-subjects T-scores ([Z scores × 10] +50) using analyses of variance (ANOVA). Startle magnitudes were averaged with each of the two habituation periods (habituation 1 and 2) (results for habituation 3 are not shown). Startle magnitudes and subjective ratings during the threat procedure were averaged across conditions, separately for cues and cue-free (i.e., ITI) periods. Alpha was set at.05 for all statistical tests. Greenhouse-Geisser corrections (GG-ε) were used for main effects and interactions involving factors with more than two levels. Pearson correlations were employed for correlation analyses.

## Results

### Startle

**Figure 2:**
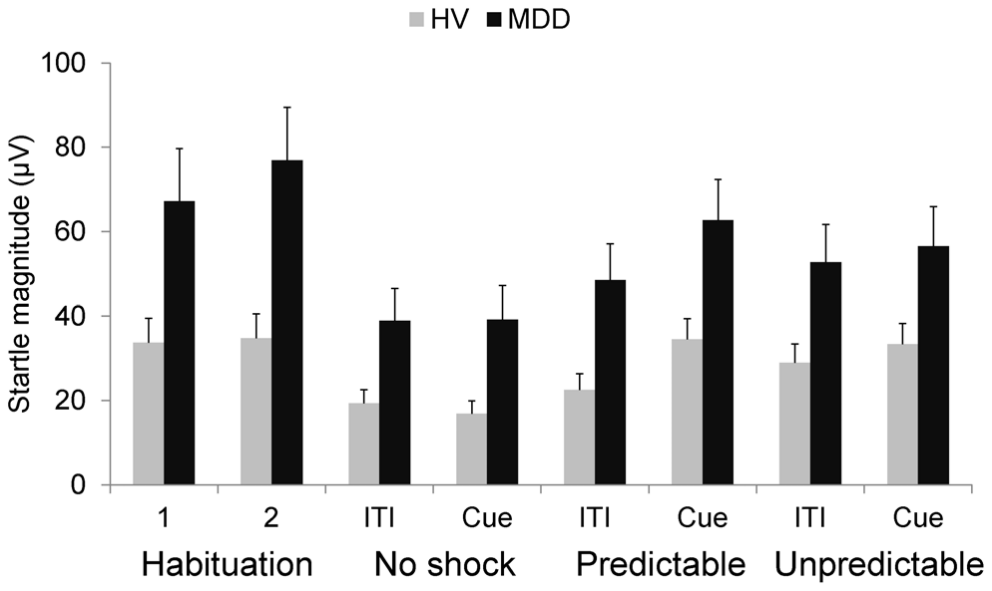
Startle response during NPU (raw scores). Startle magnitudes (raw scores) in the controls and patients with major depressive disorder (MDD) during the habituation procedures and during the no shock (N), predictable shock (P), and unpredictable shock (U) condition in the threat procedures. Error bars are SEM.

Given that previous studies have reported either normal or reduced startle responses in startle reactivity in MDD (see Introduction), we reasoned that if the MDD patients were sensitive to contextual anxiety (context-potentiated startle), this would be reflected as an overall elevated startle reactivity during the habituation procedures and/or during the subsequent NPU threat test. We therefore conducted two analyses with the raw magnitude scores, one during habituation and the other during the NPU threat test. The habituation data were examined with a Group (controls, MDD) × Sex (male, female) × Time (habituation 1, habituation 2) ANOVA. The startle habituation 1 data of one MDD subject was corrupted and could not be analyzed. This subject was excluded from the startle habituation analysis. Results showed a significant group main effect (F(1,51) = 7.0, p<.01), due to larger overall startle magnitude in the patients compared to the controls (Fig. 2) and probably reflecting increased contextual anxiety (see Discussion). The NPU threat data were entered into a Group (controls, MDD) × Sex (male, female) × Condition (N, P, U) × Stimulus Type (ITI, cue) ANOVA. Results showed a significant Group main effect (F(1,52) = 5.5, p<.02), also due to larger overall startle magnitude in the patients compared to the control. There was no significant interaction with Group or Sex in these analyses.

**Figure 3:**
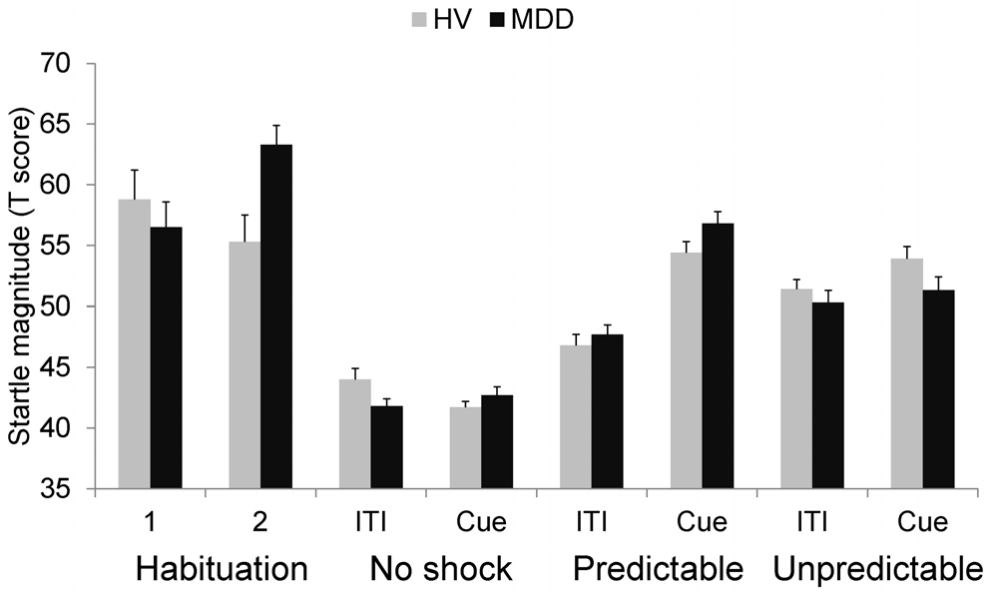
Startle response during NPU (T scores). Startle magnitudes (T scores) in the controls and patients with major depressive disorder (MDD) during the habituation and threat procedures. Error bars are SEM.

Subsequent analyses were conducted with T-scores to eliminate these large group differences in baseline startle magnitude while preserving individual patterns of differential startle responding across conditions [38]. Analysis of the habituation data (Fig. 3) revealed a significant group × time interaction (F(1,51) = 6.2, p<.02), reflecting a group difference in startle reactivity after the placement of the shock electrodes. Specifically, startle increased significantly between habituation 1 and 2 in MDD (F(1,26) = 9.3, p<.005), reflecting heightened contextual anxiety, whereas startle was unchanged during the same period in the controls (F(1,27) = .5, ns).

An omnibus Group (controls, MDD) × Sex (male, female) × Condition (N, P, U) × Stimulus Type (ITI, cue) ANOVA of the threat data show no significant interaction with Group. Consistent with our past work with this paradigm, we conducted a priori analyses of fear-potentiated startle and anxiety-potentiated startle [30], [31]. Prior to the group comparison analysis we also conducted analyses only in the controls to insure that the expected effects were obtained. Fear-potentiated startle was defined as the increased in startle magnitude from ITI to the threat cue in P (Fig. 3). As expected, in the controls startle magnitude was larger during the cue compared to ITI in P (t(27) = 8.33, p<.0009). A Group (2) × Sex (males, females) × Stimulus Type ANOVA showed a significant Stimulus Type effect (F(1,54) = 115.9, p<.0009) without Group × Stimulus Type interaction (F(1,54) = .9, ns).

Anxiety-potentiated startle was defined as the increase in ITI startle magnitude form N to P to U (Fig. 3). In the controls, startle increased linearly from the N to the U to the P condition (main effect, F(2,54) = 25.4, p<.0009, ε = .94; linear trend, F(1,27) = 39.4, p<.0009). Startle magnitude was larger during P compared to N (F(1,27) = 5.5, p<.02) and greater during U compared to P (F(1,27) = 32.0, p<.0009). A group comparison was conducted with a (Group (controls, MDD) × Sex (males, females) × Condition (N, P, U)) ANOVA. There was a significant Condition main effect (F(2,104) = 50.1, p<.0009, ε = .99; linear trend, F(1,52) = 91.4, p<.0009), a trend for significant Group × Condition interaction (F(2,104) = 2.8, p = .06), and a significant Group × Condition interaction quadratic trend (F(1,52) = 4.5, p<.03), which was due to larger startle potentiation in MDD in the predictable condition compared to the no shock condition (F(1,54) = 5.6, p<.02).

### Comorbid Anxiety Disorder

The above analyzes were conducted within the MDD group to examine the effect of comorbidity with anxiety disorder (the variable Group was MDD with comorbid anxiety (MDD_anx_) vs. MDD without comorbid anxiety (MDD_noanx)_). Baseline startle during habituation did not differ significantly between the two groups (MDDanx, F(1,25) = .001, ns; mean habituation = 73.4 µV and 72.8 µV in the MDDnoanx and MDD anx groups, respectively). Startle reactivity following the placement of the shock electrodes increased from 56.3 to 62.7 t-scores in the MDDanx group and from 52.8 to 64.0 t-scores in the MDDnoanx group. This startle potentiation was no significantly different between the two groups (F(1,25) = .7, ns). Finally, the presence of comorbid anxiety did not significantly affect fear-potentiated startle or anxiety-potentiated startle (all p>.4). These results show no effect of comorbid anxiety disorder.

### Correlations

In the patients, correlations were conducted between illness duration (time since illness; note that illness duration was missing for 2 patients), trait anxiety, BDI scores, and ITI startle magnitudes. Results showed several positive correlations among these measures (Table 1). Specifically, illness duration and BDI were significantly correlated with startle magnitudes. BDI did not correlate significantly with illness duration. To further elucidate whether BDI and illness duration contributed independently to startle reactivity, a multiple regression analysis was conducted. We calculated a mean startle score (startle_m_) over all startle responses (hab 1, hab2, and ITI and Cue during N, P, and U) to use as a dependent variable (Table 1). A regression analysis was conducted with startle_m_ as the dependent variable and BDI and illness duration as independent variables. The overall regression model was significant (F(2,23) = 5.7, p<.01). It explained 33% of the variance in startle_m_ (R_squared_ = .33), with both variables contributing significantly to the model (p = .02 and p = .05, respectively). Note that there was no significant correlation between trait anxiety and startle magnitude in the controls.

**Table 1:** Correlation (probability) between illness duration, trait anxiety, and BDI, and startle reactivity in the MDD patients.

		Illness duration	Trait anxiety	BDI
**Questionnaires**	**Trait anxiety**	.01 (.95)		
	**BDI**	.10 (.63)	.17 (.39)	
**Habituation (raw scores)**	**1**	.62 (.001)	.04 (.81)	.23 (.23)
	**2**	.56 (.003)	.16 (.40)	.31 (.10)
**ITI startle magnitude (raw scores)**	**No shock**	.43 (.01)	.16 (.40)	.40 (.03)
	**Predictable shock**	.47 (.01)	.20 (.30)	.34 (.08)
	**Unpredictable shock**	.45 (.02)	.17 (.37)	.32 (.10)
	**Startle_m_** *	.46 (.01)	.18 (.34)	.35 (.10)
**Difference T-scores**	**hab2 minus hab1**	.02 (.90)	−.24 (.22)	.36 (.06)
	**P - N**	.12 (.54)	.24 (.63)	−.12 (.54)

* Average of ITI startle magnitudes in the no-shock, predictable, and unpredictable condition.

### Subjective Anxiety

The subjective anxiety results (Table 2) were analyzed with the same ANOVA used for the startle data analysis (i.e., Group (2) × Sex (male, female) × Condition (3) × Stimulus Type (2)). The MDD group reported significantly higher anxiety compared to the control throughout the experiment (main Group effect, F(1,51) = 12.1, p<.001). There was no interaction involving the factor Group.

**Table 2:** Mean (sem) Retrospective subjective ratings of fear and anxiety.

	No shock		Predictable		Unpredictable	
	ITI	Cue	ITI	Cue	ITI	Cue
HV	1.50 (.17)	1.76 (.23)	3.60 (.39)	4.8 (.47)	4.8 (4.5)	4.02 (.49)
MDD	3.95 (.46)	3.38 (.36)	5.16 (.47)	6.1 (.48)	6.2 (.45)	5.5 (.54)

Like for the startle data, subsequent analyses were conducted with T-scores to eliminate the overall group difference in anxiety rating. The Group (controls, MDD) × Sex (male, female) × Condition (N, P, U) × Stimulus Type (ITI, cue) ANOVA of the retrospective rating show no significant interaction with Group. Comparison of retrospective anxiety ratings during ITI and the threat cue in P (Group (2) × Sex (2) × Stimulus Type (2)) show a significant Stimulus Type effect (F(1,51) = 40.5, p<.0009), due to higher anxiety ratings during the threat cue. Comparison of retrospective anxiety ratings during ITI in each condition (Group (2) × Sex (2) × Condition (3)), revealed significant Condition effect (F(2,102) = 121.8, p<.0009) and a linear Condition effect effect (F(1,51) = 268.4, p<.0009). There was no interaction with the factor group in any of these analyses.

## Discussion

Consistent with our hypothesis, there was no evidence of blunted startle potentiation in the MDD group; anticipation of shocks led to a robust level of startle potentiation in the MDD patients. In fact, there were several instances of increased startle reactivity in the patients compared to the controls. Indeed, the MDD group showed 1) overall elevated startle reactivity, 2) potentiation of startle following the placement of the shock electrodes that was not seen in the controls, and 3) increased anxiety-potentiated startle (i.e., increased ITI startle) in the predictable condition compared to the controls. These findings do not support a strong version of the ECI hypothesis that assumes a generalized blunting of defensive responses. Rather, they set limit to the ECI concept and provide evidence that MDD is associated with heightened defensive reactivity when facing an actual danger.

There are two possible explanations for the heightened baseline startle reactivity in MDD. It could reflect a chronic symptom of MDD, either as a consequence of an innate vulnerability or the disorder. At this time, there is little support for this explanation. Exaggerated startle is not a symptom of MDD, and previous studies have consistently found normal or reduced startle reactivity in MDD (see Introduction). No study has reported elevated startle in this population in innocuous contexts. The alternative explanation for the increased baseline startle in MDD is that it was a state-dependent contextually-mediated effect. Specifically, in the MDD group startle was potentiated by the threatening experimental context (context-potentiated startle), an effect we have reported in anxiety disorders. Indeed, it is well-established that aversive contexts increase startle. Startle can be potentiated by mere participation in experiments where shocks are administered and by placement of the shock electrodes [39], [40], especially in patients with anxiety disorders [28], [41] and at risk for mood disorder [32]. For example, baseline startle is normal in panic disorder in innocuous contexts [42], [43], [44], [45], but it is elevated in a threat of shock context [41]. More direct evidence for this explanation comes from a study during which PTSD veterans were tested on two separate occasions, in a threatening context (e.g., shock administration) and a non-threatening context. Baseline startle was elevated compared to non-PTSD controls only in the threatening context [28]. It is therefore highly likely that the elevated startle in MDD in the present study also reflected contextual anxiety. However, definitive evidence of increased sensitivity to contextual threat in MDD remains to be demonstrated, possibly by comparing startle reactivity in two separate sessions, one in an innocuous context and the other during a shock threat experiment.

This hypothesis is further supported by the two additional findings, which also confirm the enhanced defensive reactivity in the MDD patients. First, startle increased from before to after placement of the shock electrodes in MDD, but not in the controls. Second, the MDD patients showed heightened startle potentiation (i.e., during ITI) in the predictable condition in the MDD patients compared to the controls. In fact, this sensitivity to contextual threat may be a vulnerability marker for mood and anxiety as suggested by the fact that offspring of parents with MDD show exaggerated startle in a threatening environment [32]. The fact, that the MDD group showed increased ITI startle in the predictable condition suggests that the patients could not use this period, which signaled the absence of threat cue, as a period of relative safety to the same extent as the control. In other words, the MDD patients may not be able to use efficiently safety signals to reduce their anxiety.

While the present results parallel many of the findings in anxiety disorders [28], [30], [31], [40], [41], there were some important differences with our past results. However, there were key methodological differences among studies. In our earlier potentiated startle studies with anxious patients, we used shocks as aversive stimuli but did not use the NPU-threat test [28], [40], [41]. In our more recent studies, we used the NPU-threat test but with milder aversive stimuli, a combination of screams, loud noises, and airblasts to the neck [30], [31]. As we have reported previously [42], such stimuli do not evoke strong contextual anxiety. The results of these studies can be summarized as follow; (1) With shocks, patients with PTSD or panic disorder show increased contextual anxiety (due to the threatening context and shock electrodes); (2) In NPU-threat with milder aversive stimuli, patients with panic disorder, PTSD, but not generalized anxiety disorder, show increased anxiety-potentiated startle in the unpredictable condition. In the present study, we found increased contextual anxiety in MDD (consistent with (1)) and increased anxiety-potentiated startle in the predictable condition but not the unpredictable condition. One potential explanation for the lack of excessive anxiety-potentiated startle in MDD is that both context-potentiated startle and anxiety-potentiated startle are mediated by the same neural system. If so, the neural system responsible for anxiety-potentiated startle may have already been partially activated by contextual threat, leaving limited room for further increase in neural activation under unpredictable shock threat. Alternatively, distinct neural systems may underlie contextual anxiety and anxiety to unpredictable shocks, and MDD is overly sensitive only to the former but not the latter. Given our restricted knowledge of mechanisms underlying aversive responses to contextual and unpredictable threats, it may be too premature to argue strongly for any of these hypotheses. However, supporting the first hypothesis (single mediating mechanism) is the finding that the BNST is involved in startle potentiation evoked by both contextual and unpredictable threat [15]. The alternative interpretation entails that MDD is not sensitive to unpredictable threat, a conclusion that runs counter to the fact that unpredictable stressors are used as experimental models of depression [46].

It has been argued that the ECI hypothesis is consistent with an evolutionary concept of depression [5], as an adaptive response signaling the need to disengage from the environment [24]. In animal models of depression, such as learned helplessness and social defeat, chronic stress leads to withdrawal from the environment, motor retardation, and lack of motivation [47]. A similar argument has been put forth to explain the blunting of startle potentiation during aversive imagery in mood and anxiety disorders, which has been attributed to a lack of amygdala recruitment [23]. However, this is may not be a totally correct interpretation of the animal literature. While learned helplessness can lead to behavioral deficits and reduced motivation to engage with the environment, these symptoms should not be taken as an indication of reduced defensive responses or reduced amygdala activation [48], [49], [50]. On the contrary, in animal models, chronic stress sensitizes limbic structures and facilitates defensive reactivity [50], [51], [52].

How then can we reconcile the ECI hypothesis and findings of blunted startle potentiation in depression by others [16], [17], [18], [19], [20] with the current result of increased defensive response? Differences in the methods to induced aversive states must be considered. Specifically, blunting in depression has been obtained during IAPS pictures, films, or emotional imagery [14], [16], [23]. In these procedures, the threat is mild, hypothetical, and/or may lack personal relevance. It may be adaptive for depressed individuals to disengage from these types of threats [24]. However, it may be more difficult or even maladaptive to disengage from a real danger such as a shock [25]. The present study, as well as past results using fear conditioning with shock as unconditioned stimulus [12], show that stimuli that can cause physical harm lead to exaggerated defensive response in MDD. We propose that symptoms of disengagement from the environment and heightened defensive reactivity coexist in MDD, and that both symptoms are similarly affected by the severity and chronicity of the disorder. Indeed, while previous studies found blunted startle potentiation during IAPS pictures or emotional imagery to be associated with illness severity and chronicity [14], [16], [23], the present study shows that increase in illness duration and in BDI scores independently predicted heightened startle reactivity. Thus, MDD patients with the most enduring dysfunction and severe symptomatology show the greatest increase in defense mobilization when confronted to an actual physical threat, but show blunted startle potentiation when dealing with hypothetical and non-personal threats. A better understanding of the nature of stimuli that engage one or the other type of response will enhance our understanding of MDD.

In addition to these conceptual implications, the present results may have clinical implications. From a treatment perspective, while the ECI model suggests that seeking to reduce defensive activation in MDD may not be an optimal treatment approach [14], the present results argue against this position; therapeutic intervention with psychological (e.g., exposure therapy) or pharmacological treatments should attempt to minimize defensive activation. Sustained anxiety is mediated by activation of corticotrophin-releasing factor (CRH) receptors in the BNST [15] and CRH influence on limbic structures has been implicated in MDD [53]. Enhanced CRH activation in MDD could result from a disinhibition of CRH release from the hypothalamic paraventricular nucleus because of weak medial prefrontal cortical control [54] and/or increased expression in the amygdala [55]. Targeting the CRH system may be a valuable approach [56]. The results also have potential diagnostic implications, especially for a brain-based nosology [57]; enhanced context-potentiated startle may index a dysfunction that cuts across diagnostic boundaries [41], [58], [59], [60].

To summarize, the results are more consistent with the negative potentiation hypothesis than the ECI hypothesis. We propose that depression is associated with blunted emotional responses when confronted with mild or hypothetical and personally-irrelevant threats [5], [23]. However, in the face of an actual physical threat, MDD patients exhibit enhanced defensive reactivity. Studies in animals and in humans are beginning to identify psychopharmacological and neural mechanisms involved in contextual anxiety [15], [61]. The current experimental paradigm is a valuable translational tool to identify brain dysfunction in mood and anxiety disorders and may help uncover novel pathophysiology-based treatments.
